# Cytokine profiling and transcriptomics in mononuclear cells define immune variants in Meniere Disease

**DOI:** 10.1038/s41435-024-00260-z

**Published:** 2024-02-23

**Authors:** Marisa Flook, Elena Rojano, Alvaro Gallego-Martinez, Alba Escalera-Balsera, Patricia Perez-Carpena, M. del Carmen Moleon, Rocio Gonzalez-Aguado, Victoria Rivero de Jesus, Emilio Domínguez-Durán, Lidia Frejo, Juan A. G. Ranea, Jose Antonio Lopez-Escamez

**Affiliations:** 1 Otology and Neurotology Group CTS495, Division of Otolaryngology, Department of Surgery, Instituto de Investigación Biosanitaria, ibs.GRANADA, Granada, Universidad de Granada, Granada, Spain; 2 Sensorineural Pathology Programme, Centro de Investigación Biomédica en Red en Enfermedades Raras, CIBERER, Madrid, Spain; 3 UCL Ear Institute, University College London, London, UK; 4 Department of Molecular Biology and Biochemistry, Faculty of Sciences, University of Malaga, Malaga, Spain; 5 Institute of Biomedical Research in Malaga (IBIMA-Plataforma BIONAND), Malaga, Spain; 6 Department of Otolaryngology, Instituto de Investigación Biosanitaria, ibs.Granada, Hospital Universitario Virgen de las Nieves, Granada, Spain; 7 Department of Otolaryngology, Hospital Universitario San Cecilio, Granada, Spain; 8 Department of Otorhinolaryngology, Hospital Universitario Marques de Valdecilla, Santander, Spain; 9 Hospital Clínic, Department of Otorhinolaryngology, Barcelona, Spain; 10 Unidad de Gestión Clínica de Otorrinolaringología, Hospital Universitario Virgen Macarena, Sevilla, Spain; 11 Meniere Disease Neuroscience Research Program, Faculty of Medicine & Health, School of Medical Sciences, The Kolling Institute, University of Sydney, Sydney, NSW Australia; 12 Centro de Investigación Biomedica en Red de Enfermedades Raras (CIBERER), Instituto de Salud Carlos III, 29029 Madrid, Spain; 13 Spanish National Bioinformatics Institute (INB/ELIXIR-ES), 08034 Barcelona, Spain

**Keywords:** Gene expression, Translational immunology

## Abstract

Meniere Disease (MD) is a chronic inner ear disorder characterized by vertigo attacks, sensorineural hearing loss, tinnitus, and aural fullness. Extensive evidence supporting the inflammatory etiology of MD has been found, therefore, by using transcriptome analysis, we aim to describe the inflammatory variants of MD. We performed Bulk RNAseq on 45 patients with definite MD and 15 healthy controls. MD patients were classified according to their basal levels of IL-1β into 2 groups: high and low. Differentially expression analysis was performed using the ExpHunter Suite, and cell type proportion was evaluated using the estimation algorithms xCell, ABIS, and CIBERSORTx. MD patients showed 15 differentially expressed genes (DEG) compared to controls. The top DEGs include *IGHG1* (*p* = 1.64 $\times$ 10^−6^) and *IGLV3-21* (*p* = 6.28 $\times$ 10^−3^), supporting a role in the adaptative immune response. Cytokine profiling defines a subgroup of patients with high levels of IL-1β with up-regulation of *IL6* (*p* = 7.65 $\times$ 10^−8^) and *INHBA* (*p* = 3.39 $\times$ 10^−7^) genes. Transcriptomic data from peripheral blood mononuclear cells support a proinflammatory subgroup of MD patients with high levels of *IL6* and an increase in naïve B-cells, and memory CD8^+^ T cells.

## Introduction

Meniere Disease (MD, MIM 156,000) is a rare chronic inner ear syndrome, characterized by sensorineural hearing loss (SNHL), vertigo attacks, aural fullness, and tinnitus [1]. The condition is multifactorial, including genetic and epigenetic factors [2–4]. MD is a highly heterogeneous disease, hence various classifications of this syndrome have been reported according to clinical history [5–7], radiological findings [8], and molecular subtypes [9].

There is extensive evidence supporting the autoimmune/autoinflammatory etiology of MD [10], namely the high prevalence of diseases in this spectrum [11–13], the existence of variants in immune-related genes associated with disease progression in MD [3], and increased cytokine levels in MD patients [9, 14–17].

Previous expression studies in MD have involved targeted gene expression [18, 19] or small case series [9, 20, 21]. Shew et al. [19] identified a downregulated miRNA in MD patients’ serum and perilymph linked to inflammatory and autoimmune pathways, using a miRNA array for the study of 5 MD patients and 5 controls. Chen et al. [20] performed RNA sequencing on the intact vestibular system of 5 delayed endolymphatic hydrops/ delayed MD patients, and Sun et al. [21] studied the transcriptome of peripheral blood mononuclear cells (PBMC) from three unilateral MD type 1 patients. Despite the low sample size, both found the involvement of immune factors in the disease pathogenesis. Frejo et al. [9] identified two different subgroups of MD patients according to cytokine levels in PBMC supernatant, namely IL-1β. Moreover, these patients showed a differential gene expression profile in genes related to immune diseases and inflammation.

The gene expression profile of PBMC could indicate the molecular features of the immune cells in the affected systems in MD. We hypothesize that MD patients with high levels of IL-1β may have an autoinflammatory background. Thus, we aim to describe autoimmune/autoinflammatory variants of MD through PBMC transcriptome analysis.

## Materials and methods

### Patient recruitment

We included a total of 45 patients with definite MD and 15 healthy controls that were recruited between February 2018 and June 2021, from Spanish referral centers for MD. Patients were diagnosed according to the diagnostic criteria of the Barany Society for MD [1]. Patients with another associated otological disease or any other cause that could mimic MD and patients under immunosuppressor or antihistaminic treatment were excluded from this study. Individuals were considered healthy controls if they presented no history of hearing loss nor vestibular symptoms and were not under corticosteroid medication. The experimental protocols of this study were approved by the Institutional Review Board in all participating hospitals and every patient signed a written informed consent. The study was carried out according to the principles of the Declaration of Helsinki revised in 2013 for investigation with humans.

Patients with IL-1β levels superior to 4 pg/mL in PBMC supernatant were considered MD patients with high levels of cytokines (MDH), and patients with IL-1β levels inferior to 4 pg/mL in PBMC supernatant were considered MD patients with low levels of cytokines (MDL), according to in house measures in a set of 90 healthy individuals [22]. Therefore, of the 45 patients recruited for the study, 9 patients were MDH, and 36 patients were MDL. Sample group sizes were based on the recommendations in the work of Schurch et al. [23].

### Clinical data

A descriptive analysis was conducted using R studio for all clinical data. Patients were classified according to cytokine levels and clinical variables were compared between both groups and controls by applying Fisher’s Exact Test for qualitative variables and Student’s t-test for the quantitative variables. The level of significance considered was *p* < 0.05.

### RNA extraction

Peripheral blood samples were obtained and peripheral blood mononuclear cells (PBMCs) were isolated as previously described elsewhere [24]. RNA was extracted from approximately 8 million PBMCs per sample using High Pure RNA Isolation Kit (Hoffmann-La Roche, Switzerland, #11828665001) or NZY Total RNA Isolation kit (NZYtech, Portugal, #MB13402), following the manufacturer’s instructions. RNA concentration and quality were verified by Nanodrop (Thermo Fisher Scientific, Massachusetts, USA) and Agilent 2100 Bioanalyzer (Agilent Technologies, California, USA). The minimum quality parameters considered were a concentration superior to 20 ng/µL, a 260/280 and 260/230 ratio superior to 1.8, RIN superior to 6.8, and no degradation or contamination.

### Bulk RNA sequencing (RNAseq)

The total RNA from 60 samples were sequenced to a minimum of 40 million 150 bp paired-end reads (12 Gb) per sample. Library preparation was performed using the NEBNext® UltraTM Directional RNA Library Prep Kit (New England BioLabs, Massachusetts, USA). RNA-seq was performed on a Novaseq 6000 (Illumina, California, USA) at the Novogene Cambridge Science Park (UK) installations.

The data files from the Novaseq 6000 sequencing platform are transformed to sequence reads by CASAVA base recognition (Base Calling). RSEM [25] software package was used for estimating gene and isoform expression levels. The FASTQ files (one per sample) were pre-processed with BBTools [26] to remove adapters as described in the sequencing library documentation, to trim low-quality regions (discarding reads of quality lower than 26) and selecting reads with a minimum length of 135 nucleotides. Reads were aligned to the GRCh38 reference human genome assembly using STAR [27] (version 2.5).

### Differential expression analysis and functional analysis

Differential expression analysis was performed using the ExpHunter Suite Bioconductor package [28], which used DESeq2 and edgeR packages. Genes are labeled as prevalent or possible differentially expressed genes (DEG), based on package results: if a gene is detected as differentially expressed by the two packages it is considered a prevalent DEG. On the other hand, if a gene is detected as differentially expressed by only one, it is considered a possible DEG. A gene is considered differentially expressed if it presents an adjusted *p* < 0.05 and absolute logFC ≥1. The comparisons for this study were: MD patients versus controls, MDL patients versus controls, MDH patients versus controls, and MDL versus MDH patients.

ExpHunter Suite performs score integration to obtain combined logFC and adjusted *p* value/FDR values for each gene across all packages. The functional analysis module of ExpHunter Suite was used to search for enrichment of sets of functionally related genes, which integrates Gene Ontology and KEGG using clusterProfiler [29]. Differential transcript usage (DTU) was analyzed with satuRn [30] package and post-processing of results was performed with stageR [31] package. Transcription factors (TFs) linked with gene enrichment were analysed with GeneCodis 4 [32].

xCell [33], ABIS [34] and CIBERSORTx [35] are computational methods used to estimate the individual cell type abundance from bulk RNA sequencing data from PBMC. Evaluation of cell type proportion was performed with the estimation algorithms xCell, ABIS, and CIBERSORTx. For CIBERSORTx analysis, we used the LM22 signature matrix with TPM gene expression matrix. Cell proportions were compared between groups performing a Mann–Whitney U test. The level of significance considered was *p* < 0.05. Differences in cell proportion were considered true if found by at least 2 methods.

## Results

### MD patients have DEGs enriched in immune response

For the RNAseq data, we first checked the quality of reads after trimming (Supplementary Fig. 1). We observed that after removal of adapters and contaminants, we retained most of the readings to proceed with their alignment. We also confirmed if the alignment using STAR was correctly performed. In Supplementary Fig. 2, we can observe that for each sample a large number of reads was aligned against genes (blue boxes). With these considerations, we performed the differential expression analysis of the samples.

We evaluated the transcriptomic differences between MD patients and healthy controls. When we first run ExpHunter Suite with all samples for the MD patients against controls comparison, we observed in the data quality analysis that patient samples MD01, MD02, MD03 and MD04 were more related to the control samples in the principal component analysis (PCA), and control samples C11, C09 and C10 were more related to the patient samples, indicating possible errors in the sequencing process (Supplementary Fig. 3). After different tests in which we compared the distribution of samples in the PCA we discarded MD01, MD02, MD04, C11, C09, C10 from our analysis. We decided to keep MD03 as it was more related to other patient samples in the PCA (Supplementary Fig. 4). Once we removed these samples from our analysis, we observed in Supplementary Fig. 4 a certain mixing between controls and MD patient samples.

Finally, our study included 42 MD patients and 12 controls with a mean age of 58.09 ± 13.01 and 46.33 ± 14.81 years, and a female percentage of 57.14% and 33.33%, respectively. No clinical differences were found between MDH and MDL patients (Table 1).

**Table 1:** Clinical and demographic features of Meniere Disease patients.

	MDH (*N* = 8)	MDL (*N* = 34)	*p*
Sex (% woman) (N)	50% (4)	59% (20)	0.7061
Age (mean ± SD)	58.5 ± 6.6	58.0 ± 14.2	0.8832
Age of onset (mean ± SD)	44.5 ± 13.2	45.02 ± 13.2	0.9208
Years of evolution (mean ± SD)	14.0 ± 10.3	12.9 ± 7.8	0.7852
Laterality (% unilateral) (N)	75% (6)	74% (25)	1.0000
Affected ear (% right) (N)	25 (2)	29 (10)	0.8822
Family history of ear affections (% yes) (N)	38% (3)	59% (20)	0.4330
Family history of MD (% yes) (N)	25% (2)	15% (5)	0.6012
Headache (% yes) (N)	38% (3)	50% (17)	0.6997
Migraine (% yes) (N)	38% (3)	29% (10)	0.6861
History of Autoimmune disease (% yes) (N)	38% (3)	29% (10)	0.6861
Number of crises in last 6 months (mean ± SD)	2.0 ± 3.3	1.8 ± 2.7	0.8554
Hearing stage (%) (N)			0.2398
1	20% (1)	17% (5)	
2	40% (2)	31% (9)	
3	0% (0)	38% (11)	
4	40% (2)	14% (4)	
AAO-HNS Functional Scale (%) (N)			0.0968
1	67% (2)	30% (9)	
2	0% (0)	22% (6)	
3	0% (0)	22% (6)	
4	0% (0)	19% (5)	
5	0% (0)	7% (2)	
6	33% (1)	0% (0)	
Tumarkin crisis (% yes) (N)	25% (2)	12% (4)	0.0562
Clinical MD group (%) (N)			0.6635
Metachronic/ Classic (type 1)	43% (3)	39% (13)	
Synchronic/ Delayed (type 2)	0% (0)	0% (0)	
Familiar History (type 3)	0% (0)	15% (5)	
Migraine (type 4)	14% (1)	21% (7)	
Autoimmunity (type 5)	43% (3)	24% (8)	

*MDH* Meniere Disease patients with high levels of cytokines, *MDL* Meniere Disease patients with low levels of cytokines, *SD* standard deviation.

**Fig. 1:**
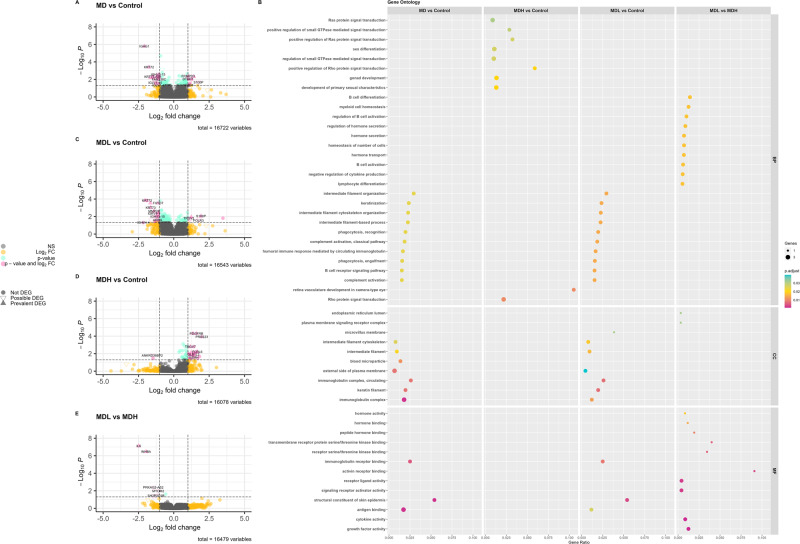
Differentially expressed genes in Meniere disease patients and controls. **A** Volcano plot of possible and prevalent differentially expressed genes (DEG) in Meniere Disease patients and controls. In pink can be found DEG with log_2_FC > 1 and *p* < 0.05; in green DEG with *p* < 0.05; in yellow DEG with log_2_FC > 1; in gray non-significant DEG. **B** Volcano plot of possible and prevalent differentially expressed genes (DEG) in MDL and controls. **C** Volcano plot of possible and prevalent differentially expressed genes (DEG) in MDH and controls. **D** Volcano plot of possible and prevalent differentially expressed genes (DEG) in MDL and MDH. **E** Dot-plot representing the gene ratio and adjusted *p* value associated to the retrieved gene ontology terms for the top 15 term for biological processes, and all terms for cellular components and molecular functions, from comparing MD patients to controls, MDL patients to controls, MDH patients to controls, and MDH patients to MDL patients, with GO functional analysis.

We identified 15 DEG (Table 2) between MD and controls, of which 4 were upregulated and 11 were downregulated, namely *IGHG1* (logFC = –2.08, p.adjust = 1.63 $\times$ 10^−6^), *KRT72* (logFC = –1.76, p.adjust = 3.99 $\times$ 10^–4^), and *IGLV3-21* (logFC = –1.65, p.adjust = 6.28 $\times$ 10^–3^) were the genes with highest differential expression (Fig. 1A).

**Table 2:** Differentially expressed genes between MD patients and controls, MDL patients and controls, MDH patients and controls, and MDL and MDH patients.

Gene Symbol	Gene Name	MD vs Controls		MDL vs Controls		MDH vs Controls		MDL vs MDH	
		logFC	p.adjust	logFC	p.adjust	logFC	p.adjust	logFC	p.adjust
*ADGRG1*	Adhesion G Protein-Coupled Receptor G1	NS	NS	NS	NS	1.16	1.34E–02	NS	NS
*ANKRD55*	Ankyrin Repeat Domain 55	–1.09	3.00E–03	–1.10	3.66E–03	NS	NS	NS	NS
*AREG*	Amphiregulin	–1.23	4.76E–03	NS	NS	NS	NS	NS	NS
*C19orf84*	Chromosome 19 Open Reading Frame 84	NS	NS	NS	NS	1.47	1.32E–02	NS	NS
*CRYBG2*	Crystallin Beta-Gamma Domain Containing 2	NS	NS	NS	NS	1.34	1.32E–02	NS	NS
*DISP2*	Dispatched RND Transporter Family Member 2	NS	NS	NS	NS	1.04	1.25E–02	NS	NS
*DTHD1*	Death Domain Containing 1	1.01	9.08E–03	NS	NS	1.35	4.95E–05	NS	NS
*ENSG00000244649*	Long Intergenic Non-Protein Coding RNA 2086	NS	NS	NS	NS	1.53	7.02E–03	NS	NS
*ENSG00000255819*	KLRC4-KLRK1; Killer Cell Lectin-Like Receptor Subfamily K Member 1	NS	NS	NS	NS	1.42	1.57E–03	NS	NS
*FAM238C*	Family With Sequence Similarity 238 Member C	–1.05	9.89E–03	–1.05	1.14E–02	NS	NS	NS	NS
*FCRL6*	Fc Receptor Like 6	1.25	9.21E–03	NS	NS	1.66	6.10E–03	NS	NS
*FRMPD3*	FERM And PDZ Domain Containing 3	1.02	4.17E–03	NS	NS	NS	NS	NS	NS
*FXYD7*	FXYD Domain Containing Ion Transport Regulator 7	–1.03	4.30E–03	–1.11	2.88E–04	NS	NS	NS	NS
*FZD4*	Frizzled Class Receptor 4	NS	NS	NS	NS	1.62	7.02E–03	NS	NS
*GLB1L2*	Galactosidase Beta 1 Like 2	NS	NS	NS	NS	1.39	1.19E–02	NS	NS
*IGHG1*	Immunoglobulin Heavy Constant Gamma 1 (G1m Marker)	–2.08	1.64E–06	–2.03	1.47E–04	NS	NS	NS	NS
*IGHV3-30*	Immunoglobulin Heavy Variable 3-30	–1.13	6.67E–03	–1.19	7.03E–03	NS	NS	NS	NS
*IGLV3-21*	Immunoglobulin Lambda Variable 3-21	–1.65	6.28E–03		NS	NS	NS	NS	NS
*IL6*	Interleukin 6	NS	NS	NS	NS	NS	NS	–2.48	7.65E–08
*INHBA*	Inhibin. Beta A (Activin A. Activin AB Alpha Polypeptide)	NS	NS	NS	NS	NS	NS	–1.97	3.39E–07
*KLRC4*	Killer Cell Lectin Like Receptor C4	NS	NS	NS	NS	1.41	7.15E–03	NS	NS
*KRT72*	Keratin 72	–1.76	3.99E–04	–1.89	1.47E–04	NS	NS	NS	NS
*KRT73*	Keratin 73	–1.51	5.65E–03	–1.64	9.57E–04	NS	NS	NS	NS
*KRT73-AS1*	KRT73 Antisense RNA 1	–1.48	4.99E–03	–1.65	2.88E–04	NS	NS	NS	NS
*LINC00239*	Long Intergenic Non-Protein Coding RNA 239	–1.05	5.36E–03	–1.07	7.83E–03	NS	NS	NS	NS
*MMP28*	Matrix Metallopeptidase 28	NS	NS	–1.41	2.43E–03	NS	NS	NS	NS
*PDGFRB*	Platelet Derived Growth Factor Receptor Beta	NS	NS	NS	NS	1.60	4.95E–05	NS	NS
*PRSS23*	Serine Protease 23	1.42	4.85E–03	1.24	1.13E–02	1.98	1.18E–04	NS	NS
*RHOU*	Ras Homolog Family Member U	NS	NS	NS	NS	1.13	1.34E–02	NS	NS
*S100P*	S100 Calcium Binding Protein P	NS	NS	1.92	8.84E–03	NS	NS	NS	NS
*SCUBE3*	Signal Peptide. CUB Domain And EGF Like Domain Containing 3	NS	NS	NS	NS	1.23	7.15E–03	NS	NS
*SETBP1*	SET Binding Protein 1	NS	NS	NS	NS	1.01	9.03E–03	NS	NS
*TRGV7*	T Cell Receptor Gamma Variable 7 (Pseudogene)	NS	NS	NS	NS	1.17	1.60E–03	NS	NS
*VIPR1*	Vasoactive Intestinal Peptide Receptor 1	NS	NS	–1.04	3.64E–03	NS	NS	NS	NS

*NS* non-significant.

The biologic processes associated with the identified DEG were evaluated through KEGG and gene ontology (GO). KEGG analysis identified pathways related to cell survival and growth (Supplementary Table 1). Whereas GO analysis identified pathways related to immunological processes and cytoskeleton organization (Fig. 1B).

The study of differential transcript usage (DTU) allows the identification of differences at the transcript-level, such as alternative splicing [30]. We performed DTU analysis with satuRn package to identify alterations in transcript expression between patients and controls. After post-processing of results with stageR package, which performs powerful gene-level tests while maintaining biological interpretation at transcript-level resolution, we observed no differences in transcript usage between patients and controls (Supplementary Table 2). No transcription factors were significantly associated with the DEG identified comparing MD to controls.

### MDH and MDL patients have different transcriptome profiles

Previous studies have shown that MD patients can be subgrouped according to their IL-1β levels [9] and that these patients show a different epigenetic signature [4], therefore we wanted to evaluate if these patients also presented transcriptomic differences. We observed that MDL patients had 13 DEG when compared to healthy controls (Table 2, Fig. 1C), and MDH patients had 17 DEG when compared to healthy controls (Table 2, Fig. 1D), of which only *PRSS23* gene was shared between comparisons (Fig. 1D). Moreover, we compared MDL to MDH patients, revealing 2 DEG, of which *IL-6* was the most downregulated gene in MDL (logFC = –2.48, p.adjust = 7.65 $\times$ 10^–8^) (Table 2, Fig. 1E).

When performing enrichment analysis, KEGG only retrieved significant pathways when comparing MDL to controls—the Neuroactive ligand-receptor interaction (p.adjust = 0.044).

Nevertheless, GO analysis associated various biological processes, molecular functions, and cellular components to the three analyses (Fig. 1B). Interestingly, the top terms associated with each comparison did not overlap. MDH are associated with signal transduction, MDL associated with immunoglobulin complexes, and comparing MDH to MDL hormonal secretion and B-cell activation seem to differentiate these patients.

No DTUs and no transcription factors were significantly associated with the DEG identified comparing MDH or MDL to controls, nor MDH to MDL.

### Differences in immune cell composition were found between MD and controls

**Fig. 2:**
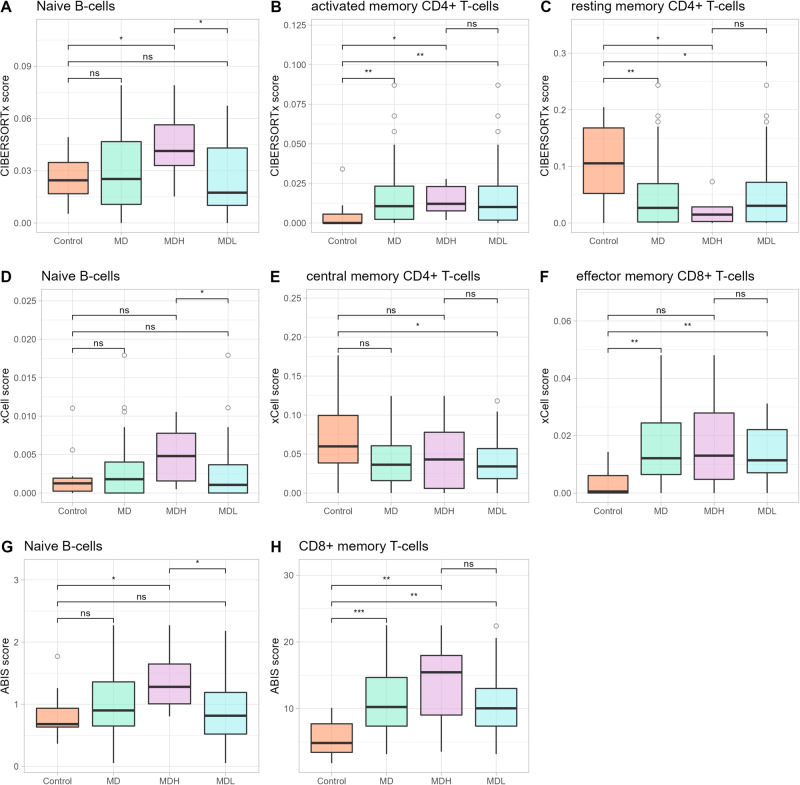
Immune cell composition inferred from bulk RNAseq deconvolution using CIBERSORTx, xCell, and ABIS. **A** Box-plot of the differences between controls, MD, MDH and MDL in naïve B-cells inferred by the CIBERSORTx score. **B** Box-plot of the differences between controls, MD, MDH and MDL in activated CD4^+^ memory T-cells inferred by the CIBERSORTx score. **C** Box-plot of the differences between controls, MD, MDH and MDL in resting CD4^+^ memory T-cells inferred by the CIBERSORTx score. **D** Box-plot of the differences between controls, MD, MDH and MDL in naïve B-cells inferred by the xCell score. **E** Box-plot of the differences between controls, MD, MDH and MDL in central memory CD4^+^ T-cells inferred by the xCell score. **F** Box-plot of the differences between controls, MD, MDH and MDL in effector memory CD8^+^ T-cells inferred by the xCell score. **G** Box-plot of the differences between controls, MD, MDH and MDL in naïve B-cells inferred by the ABIS score. **H** Box-plot of the differences between controls, MD, MDH and MDL in memory CD8^+^ T-cells inferred by the ABIS score. ns *p* > 0.05; * *p* < 0.05; ** *p* < 0.01; *** *p* < 0.001 by pair-wise Mann–Whitney U test.

We sought to evaluate if there were enriched gene signatures associated with an immune cell population in our RNAseq data. Thus, we used three deconvolution methods: CIBERSORTx, xCell and ABIS. We considered a population enriched if identified as such by at least 2 of the programs. CIBERSORTx found a positive enrichment of naïve B-cells (Fig. 2A), activated and resting memory CD4^+^ T-cells (Fig. 2B, C) (*p* < 0.05). In agreement, xCell found a positive enrichment of naïve B-cells (Fig. 2D) central memory CD4^+^ T-cells (Fig. 2E), and effector memory CD8^+^ T-cells (Fig. 2F) (*p* < 0.05). Lastly, ABIS revealed an increase in naïve B-cells (Fig. 2G), and memory CD8^+^ T-cells (Fig. 2H) (*p* < 0.05). Overall, we observed an increase in naïve B-cells in MDH patients when compared to MDL patients and controls, and a decrease in memory CD4^+^ T-cells and an increase in memory CD8^+^ T-cells in MD patients compared to controls.

## Discussion

In this work, we used bulk RNA sequencing of peripheral blood mononuclear cells from MD patients and controls to define the molecular signature of autoimmune/autoinflammatory variants of MD.

In the PCA of our data we observed low variability between samples. This could be due to low biological variability between MD patients and controls, batch effect, or sample heterogeneity, as PBMC are composed of various cell types.

Our results demonstrate that MD patients have a different transcriptomic profile than healthy controls. Moreover, we observed MD patients classified according to cytokine levels, as previously described [9] also show differing profiles between them and compared to controls, despite no clinical or demographic differences being observed. Blood samples were obtained out of the vertigo episode to generate a baseline transcriptomic profile.

Among the DEG between MD and controls the top three genes are related to the activation of the adaptative immune response and phagocytosis involving the immunoglobulin proteins P01857 (Immunoglobulin heavy constant gamma 1, *IGHG1*), P80748 (Immunoglobulin lambda variable 3-21, *IGLV3-21*) and Q14CN4 (Keratin 72, *KRT72*), probably related to non-specific changes in epithelial expression, since the genes *KRT72* and *KRT73* are also DEG when MDL are compared to controls.

MD patients show a significant decreased expression of *AREG*, encoding Amphiregulin, a member of the epidermal growth factor family which promotes the restoration of tissue integrity following damage associated with acute or chronic inflammation [36]. Moreover, AREG-gene deficient mice have impaired resolution of a variety of inflammatory challenges [36]. Thus, it is plausible that considering the role that AREG in restoring tissue integrity following infection or injury, the downregulation of this gene in MD patients could indicate a persistent inflammatory status.

*ANKRD55, FXYD7*, and *MMP28* were found downregulated in MDL patients when compared to controls. Variants in *ANKRD55* have been previously described as a risk factor for various autoimmune and inflammatory diseases, such as rheumatoid arthritis, type 1 diabetes, and inflammatory myopathies, which contrastingly to our findings are associated with higher expression of *ANKRD55* in CD4^+^ T lymphocytes [37]. *MMP28*, which is expressed by leukocytes, has been described in mice to have a role towards M2 macrophage polarization [38], and in promoting chronic inflammation and tissue remodeling, in a mouse model of exposure to cigarette smoke [39].

MD patients present endolymphatic hydrops, which have been described to be triggered by aberrant regulation of sodium by Na,K-ATPase or epithelial sodium channels [40]. Elevated dietary salt consumption increases specific inhibitors and ligands of the Na/K-ATPase potentially changing the activity of the Na,K-ATPase in cochlea. The downregulation of the *FXYD7* gene, responsible for encoding a Na,K-ATPase, has been observed in MDL. This downregulation suggests a potential association with Na,K-ATPase dysfunction, thereby contributing to the development of endolymphatic hydrops in individuals with Meniere’s disease.

*PRSS23, FCRL6, ADGRG1, KLRC4*, and *DTHD1* genes were found upregulated in MDH patients compared to controls. These genes are associated with various aspects of immune function [41], including cytotoxicity [42], NK cell maturation [42, 43], and T cell activity [44]. Their dysregulation or altered expression in Meniere’s disease patients suggests potential links to higher cytotoxic activity and immune processes involved in the development of the condition.

MD patients with high levels of IL-1β (MDH) have a persistent proinflammatory response and represent around 15-20% of cases in MD [9]. Cochlear autoinflammation and activation of NLRP3 inflammasome in vestibular-resident macrophage-like cells seems to be a common mechanism leading to chronic inflammation, in both sensorineural hearing loss [45] and MD [46].

Of note, two genes *IL6* and *INHBA* genes were upregulated in MDH patients when compared to MDL. IL-6 is a pleiotropic cytokine, with many roles in inflammation and immune response [47]. Activin A encoded by *INHBA* is described as a T_h_2 cytokine, as it is abundant in these cells, furthermore the neutralization of this cytokine in vivo significantly decreased IgE production in mice immunized with ovalbumin [48]. We have recently identified a cluster of patients with high levels of pro-inflammatory cytokines and IgE levels by mass cytometry [49]. Together, this might suggest that that differences in expression in *IL6* and *INHBA* in MDH patients might be related to a type 2 immune response.

Our deconvolution analysis of RNAseq data revealed an increase in naïve B-cells among MDH patients. Earlier studies using mass cytometry have identified elevated levels of IL-4 in MD patients with high levels of pro-inflammatory cytokines [49]. Considering that IL-4 has been demonstrated to inhibit the apoptosis of naive B lymphocytes [50], this observation suggests a potential inclination towards a type 2 immune response in MDH patients. Unfortunately, we cannot confirm this hypothesis with this data, so further experiments are needed to confirm an increased activity of T_h_2 cells in MDH patients.

Deconvolution of RNAseq data identified an increase in CD8^+^ and decrease in CD4^+^ memory T-cells in MD patients. Memory T-cells are generated after resolution of a primary response, it has been reported that the magnitude of the effector response from CD4^+^ memory T-cells correlates with the size of the resulting memory pool [51]. Notably, elevated numbers of memory CD4^+^ T-cells observed in autoimmune conditions like psoriasis suggest a potential role in promoting autoimmunity [52]. Furthermore, effector memory and resident memory CD8^+^ T-cells have been implicated in the pathogenesis of autoimmune diseases, such as multiple sclerosis and autoimmune diabetes, due to their ability to damage host tissues [53].

Previous studies suggested that patients with proinflammatory phenotype showed high levels of IL-1β, TNFα and IL-6 [9]; in our current study despite finding differences in IL-1β at a protein level, we did not observe IL-1β statistically significant changes at a transcriptomic level, but did find differences in expression of *IL6*. The differences in the results, from those previously reported [9, 19–21], may be explained by differences in the technology, sample size, tissue, and software used for the analyses, nevertheless, we have identified differences in immune response and inflammation related genes at RNA level.

Our study has some limitations. First, the sample size is small and some DEG could be missed, thus studies with a larger cohort are necessary to validate our findings. Moreover, the RNAseq technology is evolving to single cell and spatial transcriptomics that will improve the resolution at single cell level and in tissue sections. Future proteomic and transcriptomic studies at single cell level will be needed to get a better understanding of the inflammatory response in MD subgroups.

In conclusion, we found that MD patients present a different transcriptomic profile from healthy controls. MDH patients have higher expression of *IL6* than MDL patients. Furthermore, various cytotoxicity-related genes were found upregulated in MDH and may play a role in the pro-inflammatory state of these patients.

### Supplementary information

Supplementary material

## Data Availability

The datasets generated and analyzed during the current study are available in the Zenodo.org repository, under the 10.5281/zenodo.10492619.
