# Supplementary material for: Cytokine profiling and transcriptomics in mononuclear cells define immune variants in Meniere Disease
Source: Genes Immun. 2024 Feb 23;25(2):124–31. doi: 10.1038/s41435-024-00260-z (PMC11023934; doi:10.1038/s41435-024-00260-z)
Supplement: Supplementary file 1 — Supplementary material [file 41435_2024_260_MOESM1_ESM.pdf]

Supplementary Information

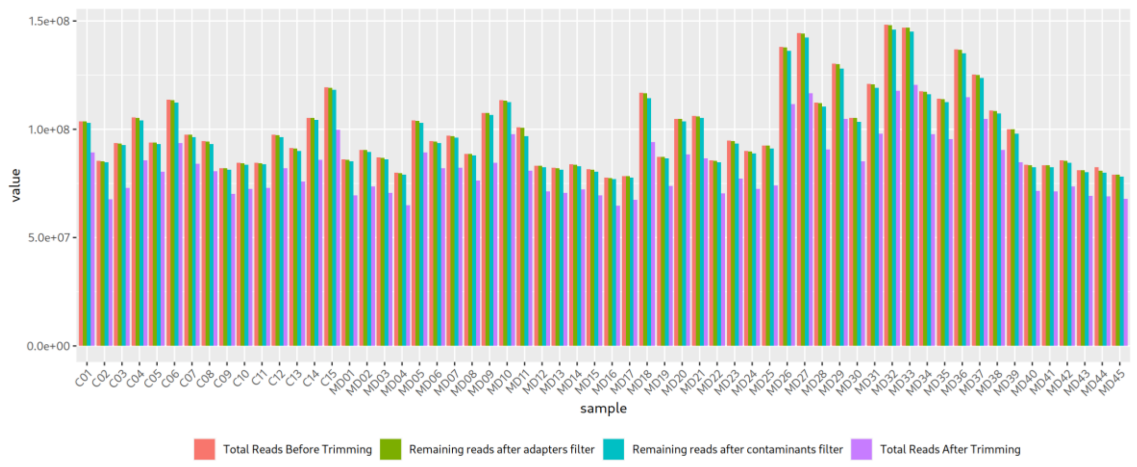

Supplementary Figure 1 - Distribution of the number of reads before trimming. Distribution of the number of reads (y axis) across the different samples (x-axis), considering the total reads before trimming (red boxes), after removing adapters (green boxes), after contaminants removal (blue box) and total reads after trimming (purple box).

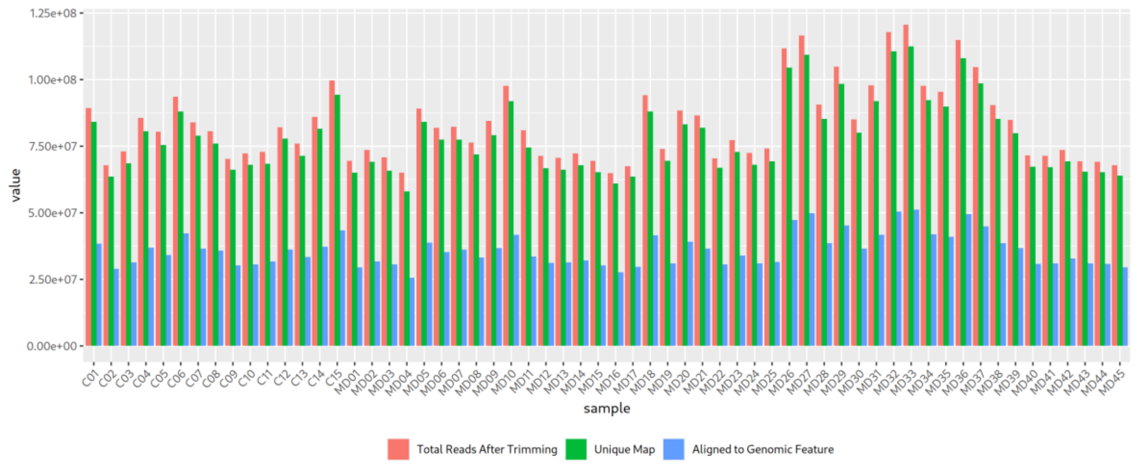

Supplementary Figure 2 - Distribution of the number of reads after trimming. Distribution of the number of reads (y axis) across the different samples (x-axis), considering the total reads after trimming (red boxes), which ones had a unique map in the reference genome (green box) and which reads aligned to a gene (blue box).

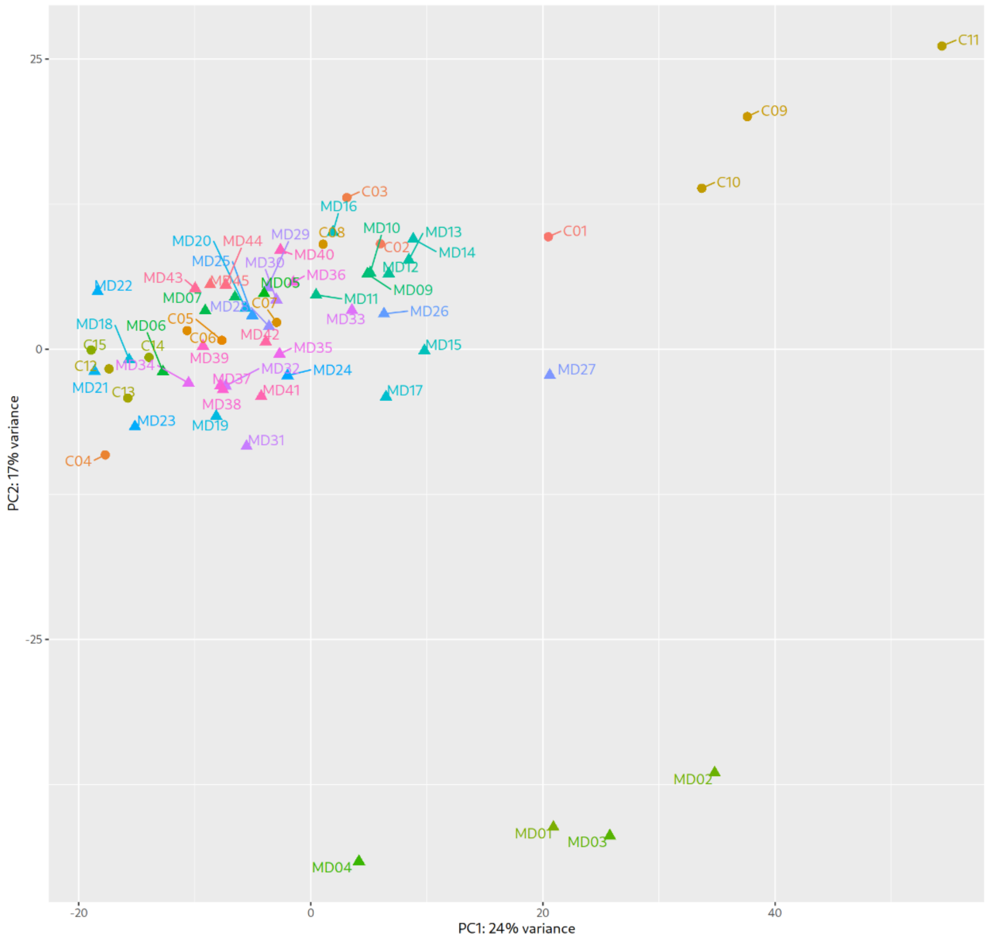

Supplementary Figure 3 - Results for principal component analysis (PCA) before sample removal. It can be observed that samples MD01, MD02, MD03, MD04 (center bottom), as well as C11, C09 and C10 (upper right corner) are very displaced with respect to the rest of the samples. Circles represent control samples and triangles represent MD patient samples.

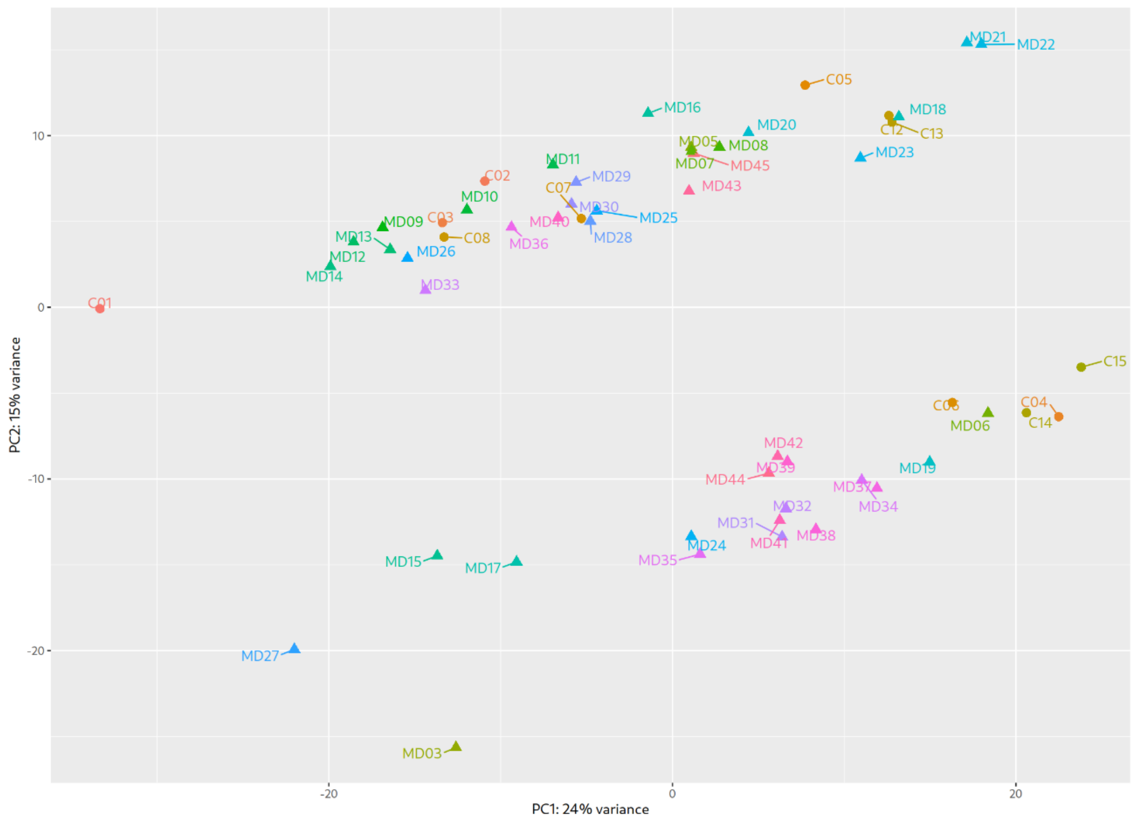

Supplementary Figure 4 - PCA without samples MD01, MD02, MD04, C11, C09 and C10. Some grouping is observed between the control samples (circles) and the MD patients (triangles), but despite this the samples have low variability between them.

Supplementary table 1 - Significative KEGG terms (adjusted p-value< 0.05) for differentially expressed genes comparing MD patients to controls. P.adjust - adjusted p-value

ID	Description	p.adjust	q-value	Gene Ratio	Gene ID
**hsa04012**	ErbB signaling pathway	0.026	NA	1/85	AREG
**hsa05210**	Colorectal cancer	0.026	NA	1/85	AREG
**hsa04390**	Hippo signaling pathway	0.032	NA	1/157	AREG
**hsa04010**	MAPK signaling pathway	0.043	NA	1/294	AREG
**hsa04151**	PI3K-Akt signaling pathway	0.043	NA	1/354	AREG

Supplementary table 2 - Differential transcript usage (DTU). txID - transcript ID; geneID - Ensmbl gene name; regular_FDR - adjusted p-value for DTU according to satuRn analysis; gene - adjusted p-value for DTU at gene level according to stageR analysis; transcript - adjusted p-value for DTU at transcript level according to stageR analysis, adjusted p-values for genes that did not pass the screening stage are by default set to NA; NA - non-aplicable

txID	geneID	MDLvControl			MDHvControl			MDLvMDH			MDvControl		
		regular_FDR	gene	transcript	regular_FDR	gene	transcript	regular_FDR	gene	transcript	regular_FDR	gene	transcript
ENST00000164640.8	ENSG00000067840.12	NA	NA	NA	0.4278	1.0000	NA	NA	NA	NA	NA	NA	NA
ENST00000229201.4	ENSG00000111602.12	0.3521	1.0000	NA	NA	NA	NA	NA	NA	NA	0.3920	1.0000	NA
ENST00000252486.9	ENSG00000130203.10	0.0146	1.0000	NA	0.2688	1.0000	NA	NA	NA	NA	0.0010	0.3140	NA
ENST00000252771.11	ENSG00000130475.14	NA	NA	NA	NA	NA	NA	NA	NA	NA	0.3920	1.0000	NA
ENST00000261741.10	ENSG00000122965.11	NA	NA	NA	NA	NA	NA	NA	NA	NA	0.3920	1.0000	NA
ENST00000261893.9	ENSG00000103642.12	0.1858	1.0000	NA	NA	NA	NA	NA	NA	NA	0.1883	1.0000	NA
ENST00000264637.8	ENSG00000126351.12	NA	NA	NA	NA	NA	NA	NA	NA	NA	0.2762	1.0000	NA
ENST00000265044.6	ENSG00000114850.6	NA	NA	NA	NA	NA	NA	0.3594	1.0000	NA	NA	NA	NA
ENST00000265586.10	ENSG00000114770.17	NA	NA	NA	0.4552	1.0000	NA	NA	NA	NA	NA	NA	NA
ENST00000272198.10	ENSG00000143847.15	NA	NA	NA	NA	NA	NA	NA	NA	NA	0.2612	1.0000	NA
ENST00000288986.6	ENSG00000158092.7	NA	NA	NA	NA	NA	NA	NA	NA	NA	0.2612	1.0000	NA
ENST00000292591.12	ENSG00000161013.17	NA	NA	NA	0.2688	1.0000	NA	NA	NA	NA	NA	NA	NA
ENST00000294984.7	ENSG00000162892.16	NA	NA	NA	0.4552	1.0000	NA	NA	NA	NA	NA	NA	NA
ENST00000302125.9	ENSG00000170476.16	0.2459	1.0000	NA	0.4552	1.0000	NA	NA	NA	NA	0.0959	1.0000	NA
ENST00000312521.9	ENSG00000174738.13	0.3276	1.0000	NA	NA	NA	NA	NA	NA	NA	0.2541	1.0000	NA
ENST00000323460.10	ENSG00000119231.11	NA	NA	NA	0.4552	1.0000	NA	NA	NA	NA	NA	NA	NA
ENST00000327761.7	ENSG00000068028.17	NA	NA	NA	0.4552	1.0000	NA	NA	NA	NA	0.2612	1.0000	NA
ENST00000338087.10	ENSG00000155926.14	NA	NA	NA	0.4278	1.0000	NA	NA	NA	NA	NA	NA	NA
ENST00000338758.11	ENSG00000188677.14	NA	NA	NA	0.4278	1.0000	NA	NA	NA	NA	NA	NA	NA
ENST00000341749.7	ENSG00000150527.17	NA	NA	NA	0.4278	1.0000	NA	NA	NA	NA	NA	NA	NA

txID	geneID	MDLvControl			MDHvControl			MDLvMDH			MDvControl		
		regular_FDR	gene	transcript	regular_FDR	gene	transcript	regular_FDR	gene	transcript	regular_FDR	gene	transcript
ENST00000345125.7	ENSG00000164733.21	NA	NA	NA	NA	NA	NA	NA	NA	NA	0.3920	1.0000	NA
ENST00000348564.11	ENSG00000081237.20	NA	NA	NA	NA	NA	NA	0.3861	1.0000	NA	NA	NA	NA
ENST00000356406.10	ENSG00000087008.16	NA	NA	NA	0.4552	1.0000	NA	NA	NA	NA	NA	NA	NA
ENST00000358290.9	ENSG00000198189.11	0.2095	1.0000	NA	NA	NA	NA	NA	NA	NA	0.1338	1.0000	NA
ENST00000360453.8	ENSG00000155846.17	NA	NA	NA	NA	NA	NA	0.3861	1.0000	NA	NA	NA	NA
ENST00000370131.3	ENSG00000137992.14	0.1858	1.0000	NA	NA	NA	NA	NA	NA	NA	NA	NA	NA
ENST00000370132.8	ENSG00000137992.14	0.1858	1.0000	NA	NA	NA	NA	NA	NA	NA	NA	NA	NA
ENST00000372077.8	ENSG00000112715.23	NA	NA	NA	NA	NA	NA	0.3861	1.0000	NA	NA	NA	NA
ENST00000372988.8	ENSG00000112576.12	0.1097	1.0000	NA	NA	NA	NA	NA	NA	NA	0.0748	1.0000	NA
ENST00000373706.9	ENSG00000121764.11	NA	NA	NA	0.4552	1.0000	NA	NA	NA	NA	NA	NA	NA
ENST00000374792.6	ENSG00000181409.14	NA	NA	NA	0.4370	1.0000	NA	NA	NA	NA	NA	NA	NA
ENST00000380464.7	ENSG00000147872.10	NA	NA	NA	0.4552	1.0000	NA	NA	NA	NA	NA	NA	NA
ENST00000381072.5	ENSG00000067057.17	NA	NA	NA	NA	NA	NA	NA	NA	NA	0.3583	1.0000	NA
ENST00000383773.8	ENSG00000174738.13	0.3276	1.0000	NA	NA	NA	NA	NA	NA	NA	0.2541	1.0000	NA
ENST00000390543.3	ENSG00000211892.4	0.0427	1.0000	NA	NA	NA	NA	NA	NA	NA	0.0086	0.8708	NA
ENST00000390545.3	ENSG00000211893.4	0.1319	1.0000	NA	NA	NA	NA	NA	NA	NA	0.0712	1.0000	NA
ENST00000390548.6	ENSG00000211896.7	0.0401	1.0000	NA	0.4278	1.0000	NA	NA	NA	NA	0.0086	0.7371	NA
ENST00000390549.6	ENSG00000211896.7	0.0401	1.0000	NA	0.4278	1.0000	NA	NA	NA	NA	0.0086	0.7371	NA
ENST00000390551.6	ENSG00000211897.9	0.0139	0.7796	NA	0.4278	1.0000	NA	NA	NA	NA	0.0023	0.6224	NA
ENST00000391929.7	ENSG00000162892.16	NA	NA	NA	NA	NA	NA	0.3861	1.0000	NA	NA	NA	NA
ENST00000392320.7	ENSG00000138378.19	0.3521	1.0000	NA	NA	NA	NA	NA	NA	NA	NA	NA	NA
ENST00000398753.5	ENSG00000182093.16	NA	NA	NA	NA	NA	NA	0.2693	1.0000	NA	NA	NA	NA
ENST00000403528.6	ENSG00000121764.11	NA	NA	NA	0.3621	1.0000	NA	0.1065	1.0000	NA	NA	NA	NA
ENST00000409236.6	ENSG00000144218.19	NA	NA	NA	0.4278	1.0000	NA	NA	NA	NA	NA	NA	NA
ENST00000409406.1	ENSG00000173933.20	0.3276	1.0000	NA	NA	NA	NA	NA	NA	NA	NA	NA	NA

txID	geneID	MDLvControl			MDHvControl			MDLvMDH			MDvControl		
		regular_FDR	gene	transcript	regular_FDR	gene	transcript	regular_FDR	gene	transcript	regular_FDR	gene	transcript
ENST00000409729.1	ENSG00000115541.11	NA	NA	NA	NA	NA	NA	0.3861	1.0000	NA	NA	NA	NA
ENST00000413507.3	ENSG00000103642.12	0.1858	1.0000	NA	NA	NA	NA	NA	NA	NA	0.1883	1.0000	NA
ENST00000418113.5	ENSG00000170264.13	NA	NA	NA	0.4278	1.0000	NA	NA	NA	NA	NA	NA	NA
ENST00000424777.6	ENSG00000147576.17	NA	NA	NA	NA	NA	NA	0.3861	1.0000	NA	NA	NA	NA
ENST00000426665.6	ENSG00000256223.6	NA	NA	NA	0.4278	1.0000	NA	NA	NA	NA	NA	NA	NA
ENST00000430553.6	ENSG00000134333.14	0.3521	1.0000	NA	NA	NA	NA	NA	NA	NA	0.2064	1.0000	NA
ENST00000436344.7	ENSG00000108244.17	0.1341	1.0000	NA	NA	NA	NA	NA	NA	NA	0.1111	1.0000	NA
ENST00000446996.5	ENSG00000130203.10	0.1080	1.0000	NA	NA	NA	NA	NA	NA	NA	0.0086	0.3140	NA
ENST00000450525.6	ENSG00000126351.12	NA	NA	NA	NA	NA	NA	NA	NA	NA	0.3233	1.0000	NA
ENST00000454079.5	ENSG00000004534.15	0.3276	1.0000	NA	NA	NA	NA	NA	NA	NA	0.3583	1.0000	NA
ENST00000463866.1	ENSG00000240344.9	0.2095	1.0000	NA	NA	NA	NA	NA	NA	NA	0.1883	1.0000	NA
ENST00000464182.5	ENSG00000100316.16	0.3521	1.0000	NA	NA	NA	NA	NA	NA	NA	NA	NA	NA
ENST00000467411.5	ENSG00000144354.14	0.2769	1.0000	NA	NA	NA	NA	NA	NA	NA	0.1592	1.0000	NA
ENST00000467705.6	ENSG00000026297.15	0.2490	1.0000	NA	NA	NA	NA	NA	NA	NA	NA	NA	NA
ENST00000468772.5	ENSG00000143398.20	0.3521	1.0000	NA	NA	NA	NA	NA	NA	NA	NA	NA	NA
ENST00000469566.5	ENSG00000183291.17	NA	NA	NA	NA	NA	NA	NA	NA	NA	0.2541	1.0000	NA
ENST00000470052.5	ENSG00000120008.16	NA	NA	NA	0.4278	1.0000	NA	0.3594	1.0000	NA	NA	NA	NA
ENST00000470347.1	ENSG00000008282.8	NA	NA	NA	0.4278	1.0000	NA	0.3594	1.0000	NA	NA	NA	NA
ENST00000474806.1	ENSG00000067208.14	NA	NA	NA	0.4278	1.0000	NA	NA	NA	NA	NA	NA	NA
ENST00000476077.1	ENSG00000099958.14	NA	NA	NA	0.4370	1.0000	NA	NA	NA	NA	NA	NA	NA
ENST00000477116.5	ENSG00000256053.7	NA	NA	NA	0.4278	1.0000	NA	NA	NA	NA	NA	NA	NA
ENST00000480614.1	ENSG00000112715.23	NA	NA	NA	0.2688	1.0000	NA	NA	NA	NA	NA	NA	NA
ENST00000481718.1	ENSG00000044446.12	NA	NA	NA	NA	NA	NA	NA	NA	NA	0.3583	1.0000	NA
ENST00000482172.5	ENSG00000032742.17	0.2459	1.0000	NA	NA	NA	NA	NA	NA	NA	0.2762	1.0000	NA
ENST00000487745.5	ENSG00000187609.16	NA	NA	NA	0.4278	1.0000	NA	NA	NA	NA	NA	NA	NA

txID	geneID	MDLvControl			MDHvControl			MDLvMDH			MDvControl		
		regular_FDR	gene	transcript	regular_FDR	gene	transcript	regular_FDR	gene	transcript	regular_FDR	gene	transcript
ENST00000490768.1	ENSG00000143224.18	NA	NA	NA	0.4278	1.0000	NA	NA	NA	NA	NA	NA	NA
ENST00000491892.1	ENSG00000128191.16	0.1377	1.0000	NA	NA	NA	NA	NA	NA	NA	0.1323	1.0000	NA
ENST00000493034.1	ENSG00000100100.13	0.0757	1.0000	NA	NA	NA	NA	NA	NA	NA	0.0524	1.0000	NA
ENST00000497139.5	ENSG00000112715.23	0.3521	1.0000	NA	NA	NA	NA	NA	NA	NA	NA	NA	NA
ENST00000503120.5	ENSG00000170476.16	NA	NA	NA	NA	NA	NA	NA	NA	NA	0.2764	1.0000	NA
ENST00000505370.1	ENSG00000197530.12	NA	NA	NA	0.4278	1.0000	NA	NA	NA	NA	NA	NA	NA
ENST00000507229.5	ENSG00000197530.12	NA	NA	NA	0.2688	1.0000	NA	NA	NA	NA	NA	NA	NA
ENST00000507518.5	ENSG00000198189.11	0.2490	1.0000	NA	NA	NA	NA	NA	NA	NA	0.1338	1.0000	NA
ENST00000509354.1	ENSG00000038382.20	0.3276	1.0000	NA	NA	NA	NA	NA	NA	NA	NA	NA	NA
ENST00000513016.5	ENSG00000038427.16	NA	NA	NA	0.4278	1.0000	NA	0.2772	1.0000	NA	NA	NA	NA
ENST00000518168.5	ENSG00000161013.17	NA	NA	NA	0.4278	1.0000	NA	NA	NA	NA	NA	NA	NA
ENST00000518548.5	ENSG00000169045.17	NA	NA	NA	NA	NA	NA	NA	NA	NA	0.2612	1.0000	NA
ENST00000518630.5	ENSG00000010810.17	NA	NA	NA	NA	NA	NA	NA	NA	NA	0.3583	1.0000	NA
ENST00000521997.5	ENSG00000123124.14	NA	NA	NA	NA	NA	NA	0.3594	1.0000	NA	NA	NA	NA
ENST00000527871.5	ENSG00000140988.16	0.2948	1.0000	NA	NA	NA	NA	NA	NA	NA	0.3872	1.0000	NA
ENST00000529070.1	ENSG00000204241.8	0.3521	1.0000	NA	NA	NA	NA	NA	NA	NA	NA	NA	NA
ENST00000531372.1	ENSG00000254709.8	0.3521	1.0000	NA	NA	NA	NA	NA	NA	NA	NA	NA	NA
ENST00000532327.1	ENSG00000204241.8	0.3521	1.0000	NA	NA	NA	NA	NA	NA	NA	NA	NA	NA
ENST00000536943.1	ENSG00000167770.11	NA	NA	NA	0.4278	1.0000	NA	NA	NA	NA	NA	NA	NA
ENST00000537866.5	ENSG00000159579.14	NA	NA	NA	0.4278	1.0000	NA	NA	NA	NA	NA	NA	NA
ENST00000539593.1	ENSG00000136003.15	0.3521	1.0000	NA	NA	NA	NA	NA	NA	NA	0.2762	1.0000	NA
ENST00000540470.5	ENSG00000133106.14	0.2095	1.0000	NA	NA	NA	NA	NA	NA	NA	0.2023	1.0000	NA
ENST00000550791.1	ENSG00000135452.10	0.2948	1.0000	NA	NA	NA	NA	NA	NA	NA	NA	NA	NA
ENST00000551150.5	ENSG00000089157.16	NA	NA	NA	0.4278	1.0000	NA	0.3079	1.0000	NA	NA	NA	NA
ENST00000555349.5	ENSG00000100650.16	NA	NA	NA	0.4278	1.0000	NA	NA	NA	NA	NA	NA	NA

txID	geneID	MDLvControl			MDHvControl			MDLvMDH			MDvControl		
		regular_FDR	gene	transcript	regular_FDR	gene	transcript	regular_FDR	gene	transcript	regular_FDR	gene	transcript
ENST00000555759.1	ENSG00000176438.12	0.1319	1.0000	NA	NA	NA	NA	NA	NA	NA	0.1111	1.0000	NA
ENST00000560359.1	ENSG00000244879.8	NA	NA	NA	0.4370	1.0000	NA	NA	NA	NA	NA	NA	NA
ENST00000560525.6	ENSG00000140332.16	NA	NA	NA	0.4278	1.0000	NA	NA	NA	NA	NA	NA	NA
ENST00000562155.5	ENSG00000104731.14	0.3276	1.0000	NA	NA	NA	NA	NA	NA	NA	NA	NA	NA
ENST00000567365.2	ENSG00000103549.21	NA	NA	NA	0.4278	1.0000	NA	0.1065	1.0000	NA	NA	NA	NA
ENST00000569748.5	ENSG00000166747.12	NA	NA	NA	0.4278	1.0000	NA	NA	NA	NA	NA	NA	NA
ENST00000572584.2	ENSG00000122390.19	0.1858	1.0000	NA	NA	NA	NA	NA	NA	NA	NA	NA	NA
ENST00000573919.1	ENSG00000173821.19	NA	NA	NA	NA	NA	NA	NA	NA	NA	0.2612	1.0000	NA
ENST00000577324.1	ENSG00000010244.18	NA	NA	NA	0.4278	1.0000	NA	NA	NA	NA	0.3583	1.0000	NA
ENST00000590633.1	ENSG00000198356.12	NA	NA	NA	0.4278	1.0000	NA	NA	NA	NA	NA	NA	NA
ENST00000590918.6	ENSG00000010310.9	NA	NA	NA	NA	NA	NA	0.3151	1.0000	NA	NA	NA	NA
ENST00000593902.1	ENSG00000181027.11	NA	NA	NA	0.2688	1.0000	NA	0.2923	1.0000	NA	NA	NA	NA
ENST00000597801.1	ENSG00000142552.8	NA	NA	NA	NA	NA	NA	0.3730	1.0000	NA	NA	NA	NA
ENST00000602320.1	ENSG00000102977.16	NA	NA	NA	NA	NA	NA	0.1065	1.0000	NA	NA	NA	NA
ENST00000604749.1	ENSG00000271109.2	NA	NA	NA	NA	NA	NA	0.1837	1.0000	NA	NA	NA	NA
ENST00000620676.6	ENSG00000048707.15	NA	NA	NA	0.4278	1.0000	NA	NA	NA	NA	NA	NA	NA
ENST00000622567.4	ENSG00000065809.13	0.2095	1.0000	NA	NA	NA	NA	NA	NA	NA	0.2252	1.0000	NA
ENST00000636315.1	ENSG00000275895.7	0.2769	1.0000	NA	NA	NA	NA	NA	NA	NA	0.1827	1.0000	NA
ENST00000638540.1	ENSG00000243335.9	NA	NA	NA	0.4278	1.0000	NA	NA	NA	NA	NA	NA	NA
ENST00000641095.1	ENSG00000211893.4	0.1319	1.0000	NA	NA	NA	NA	NA	NA	NA	0.0712	1.0000	NA
ENST00000641136.1	ENSG00000211897.9	0.0139	0.7796	NA	0.4278	1.0000	NA	NA	NA	NA	0.0023	0.6224	NA
ENST00000641146.1	ENSG00000164073.10	NA	NA	NA	0.4278	1.0000	NA	0.2693	1.0000	NA	NA	NA	NA
ENST00000641978.1	ENSG00000211892.4	0.0427	1.0000	NA	NA	NA	NA	NA	NA	NA	0.0086	0.8708	NA
ENST00000650708.1	ENSG00000183735.10	0.2769	1.0000	NA	NA	NA	NA	NA	NA	NA	NA	NA	NA
ENST00000659687.1	ENSG00000238005.4	0.0764	1.0000	NA	NA	NA	NA	NA	NA	NA	0.0430	1.0000	NA

txID	geneID	MDLvControl			MDHvControl			MDLvMDH			MDvControl		
		regular_FDR	gene	transcript	regular_FDR	gene	transcript	regular_FDR	gene	transcript	regular_FDR	gene	transcript
ENST00000673027.1	ENSG00000156110.14	NA	NA	NA	0.4370	1.0000	NA	NA	NA	NA	NA	NA	NA
